# Anthocyanin-Rich Supplementation: Emerging Evidence of Strong Potential for Sport and Exercise Nutrition

**DOI:** 10.3389/fnut.2022.864323

**Published:** 2022-03-31

**Authors:** Mark E. T. Willems, Sam D. Blacker

**Affiliations:** Institute of Sport, Nursing and Allied Health, University of Chichester, Chichester, United Kingdom

**Keywords:** blackcurrant, anthocyanins, exercise, supplementation, experimental design, bioavailability, responders

## Abstract

Dark-colored fruits, especially berries, have abundant presence of the polyphenol anthocyanin which have been show to provide health benefits. Studies with the berry blackcurrant have provided notable observations with application for athletes and physically active individuals. Alterations in exercise-induced substrate oxidation, exercise performance of repeated high-intensity running and cycling time-trial and cardiovascular function at rest and during exercise were observed with intake of New Zealand blackcurrant. The dynamic plasma bioavailability of the blackcurrant anthocyanins and the anthocyanin-derived metabolites must have changed cell function to provide meaningful *in-vivo* physiological effects. This perspective will reflect on the research studies for obtaining the applied *in-vivo* effects by intake of anthocyanin-rich supplementation, the issue of individual responses, and the emerging strong potential of anthocyanins for sport and exercise nutrition. Future work with repeated intake of known amount and type of anthocyanins, gut microbiota handling of anthocyanins, and coinciding measurements of plasma anthocyanin and anthocyanin-derived metabolites and *in-vivo* cell function will be required to inform our understanding for the unique potential of anthocyanins as a nutritional ergogenic aid for delivering meaningful effects for a wide range of athletes and physically active individuals.

## Introduction

Fruits and vegetables provide primarily the dietary intake of polyphenols. The focus for intake effects in athletes and physically active individuals has been directed toward the major class of polyphenols, i.e., the flavonoids, and the major sub-class of the flavonoids, i.e., the anthocyanins. Dark-colored berries are rich in anthocyanins. Anthocyanins provide health benefits partly by anti-inflammatory and anti-oxidant effects (1, 2). Exercise, particularly with high intensity and duration, provides cell oxidative stress and inflammation. Therefore, the anti-inflammatory and anti-oxidant effects of anthocyanins provided justification for the early studies to examine the ergogenic potential of anthocyanin intake for athletes [i.e., chokeberry (3), blackcurrant (4, 5)]. The primary aim of this perspective is to provide a reflection on the existing experimental evidence for supplementation with anthocyanins as an effective nutritional ergogenic aid in the field of sport and exercise nutrition. It will focus primarily on human studies that examined the effects by the intake of anthocyanin-rich blackcurrant on performance during exercise. The secondary aim will be to highlight the outstanding issues that need to be addressed to strengthen the knowledge base on the mechanisms of anthocyanin-rich supplementation for athletes to enhance performance during exercise and advocate design and methodological issues for future studies.

## Setting The Scene For Anthocyanin Effects In Sport And Exercise

In 2005, Matsumoto et al. (6) reported that 2–3 h after intake of anthocyanin-rich blackcurrant concentrate [17 mg·(kg body weight^−1^) containing 10.83% anthocyanins], blood flow at rest measured with near-infrared spectroscopy after venous occlusion was enhanced by 22–26% in human forearms. Blackcurrant (*Ribes Nigrum*) is rich in delphinidins [~66.1% of total anthocyanin content (TAC)] and contains mostly the anthocyanins cyanidin-3-*O*-rutinoside (~27.1% of TAC), cyanidin-3-*O*-glucoside (~4.2% of TAC), delphinidin-3-*O*-rutinoside (~51.5% of TAC) and delphinidin-3-*O*-glucoside (~14.6% of TAC) with lesser amounts of cyanidin-3-*O*-(6″-p-coumaroyl-glucoside (~0.3% of TAC), pelargonidin-3-*O-*rutinoside (~0.4% of TAC), peonidin-3-*O*-rutinoside (~0.2% of TAC), petunidin-3-*O*-(6″-p-coumaroyl-glucoside (~0.9% of TAC) and petunidin-3-*O-*rutinoside (~0.7% of TAC) [http://phenol-explorer.eu/: (7)]. In the Matsumoto et al. study on the acute intake effects, male participants (*n* = 9, age: 29.2 ± 1.1 years) were tested after an overnight fast and did not consume anthocyanin-rich food on the day before testing (6). The important role of blood flow for oxygen and nutrient delivery and removal of metabolic by-products at rest and during exercise is irrefutable (8, 9). The ability of blackcurrant to affect blood flow was also observed by Barnes et al. (10) who examined the effects of acute intake of New Zealand blackcurrant extract in healthy males [*n* = 10, average BMI: 28.1 kg·m^−2^), 1.87 mg of anthocyanins·(kg body weight)^−1^] on hemodynamics during 120 min of prolonged sedentary sitting (known to adversely affect hemodynamic responses). Similar to the study by Matsumoto et al. (6), participants in Barnes et al. (10) avoided anthocyanin-containing foods for 24 h. Forearm blood flow was measured with venous occlusion plethysmography and forearm circumference measurements (10). New Zealand blackcurrant extract abolished the decrease in forearm blood flow during prolonged sitting between 90 and 120 min (10). This observation was associated with the absence of a change in vascular resistance during prolonged sitting between 90 and 120 min. At 120 min, concentrations of nitrate (vasodilator), nitrite (vasodilator) and endothelin-1 (vasoconstrictor), were not affected (10). The observations on enhanced forearm blood flow with blackcurrant intake by Matsumoto et al. (6) and Barnes et al. (10) suggest an acute effect on hemodynamic responses, at least in forearm blood flow. In the same paper by Matsumoto et al. (6), observations were also provided for a second study on the effects of 2 weeks intake of blackcurrant concentrate [7.7 mg · (kg body weight)^−1^] on oxygen uptake and muscle activity (root-mean-square of EMG recordings) during typing work. During the 2 weeks dosing period, participants [*n* = 11 (8 males), age: 39.0 ± 11.6 years] were requested to avoid anthocyanin-rich foods such as fruits, vegetables and juices. During typing work (six sets of 5 min with 1-min rest in between sets), blackcurrant concentrate seemed to have enhanced oxygen supply (and blood flow) as muscle activity was lower. The observations by Matsumoto et al. (6) provided a rationale for future studies on the effects of anthocyanin-rich blackcurrant intake on exercise responses.

One of the earliest study on the ergogenic potential of a berry in athletes was done in male elite rowers (Polish rowing team, age ~22 years) with daily intake of 3 × 50 mL of chokeberry (*Aronia)* juice for 4 weeks (providing ~34.5 mg of anthocyanins per day) (3). Chokeberry contains primarily cyanidin-3-*O*-arabinoside (~28.8% of TAC), cyanidin-3-*O*-galactoside (~63.5% of TAC), cyanidin-3-*O*-glucoside (~2.2% of TAC), cyanidin-3-*O*-xyloside (~2.5% of TAC) and pelargonidin-3-*O*-arabinoside (~0.2% of TAC) [http://phenol-explorer.eu/: (7)]. Chokeberry juice reduced 2,000 m rowing-induced glutathione peroxidase activity by 34% (1 min in recovery) and superoxide dismutase activity by 9% (24 h in recovery) in red blood cells, suggesting adaptation with an enhanced endogenous antioxidant defense system (3). In addition, in the chokeberry juice group, there was a 39% reduction in thiobarbituric acid reactive substances, a marker of lipid peroxidation, albeit criticized now as a valid marker of oxidative stress (11). Although chokeberries have one of the highest amounts of anthocyanins with 99.8% cyanidins, the anthocyanins in chokeberry juice make up ~25% of the total polyphenols (12). Therefore, observations in Pilaczynska-Szczesniak et al. (3) may have had contribution from flavonols, flavanols, proanthocyanidins, and phenolic acids. Subsequent berry studies confirmed beneficial post-exercise recovery effects on running-induced (2.5 h at 72% $\dot{V}O_{\text{2max}}$) natural killer cells, oxidative stress and inflammation [blueberry (*Vaccinium sect. Cyanococcus*): (13)], effects on muscle damage and inflammation after a half-marathon [bilberry (*Vaccinium myrtillus)*: (14)], and New Zealand blackcurrant effects on rowing-induced (30 min at 80% $\dot{V}O_{\text{2max}}$) oxidative stress and inflammation (5). The focus of the early berry studies [e.g., (3, 5, 13)] was on the recovery of exercise-induced inflammation and oxidative stress. However, the physiological responses (e.g., enhanced blood flow) with acute and chronic intake of anthocyanin-rich blackcurrant concentrate in Matsumoto et al. (6) and the potential for antioxidant effects in the study by Lyall et al. (5) by blackcurrant intake may suggest strong potential for the effect of berry intake on responses during whole-body exercise and exercise performance.

## Anthocyanins And Exercise Performance

Cook et al. (15) used a randomized, placebo-controlled, double-blind, cross-over design and reported for the first time a berry-induced exercise performance-enhancing effect. In this study, a 16.1 km cycling ergometer time-trial was improved by 2.4% for male cyclists (range: −2.7 to 8.7% with 5 participants a change higher than 5.9%). Participants performed two full 16.1 km familiarizations and were allowed a small breakfast 3 h before testing to maintain ecological validity (15). The dosing strategy in Cook et al. (15) was 7-days intake of capsulated extract made from New Zealand blackcurrants (105 mg anthocyanins·day^−1^) with the last dose 2 h before testing. The study by Cook et al. (15) had no dietary restrictions during the 7 days of dosing. As mentioned previously, blackcurrant is rich in delphinidins. In comparison with other anthocyanins, delphinidins have the largest total number of hydroxyl groups and largest number of B-ring located hydroxyl groups providing them with potent intracellular radical scavenging activity (16). Nevertheless, Maqui (*Aristotelia chilensis*) berries also have a high delphinidin content (17, 18) but there are no studies on the maqui potential for enhancing exercise performance. The effectiveness of New Zealand blackcurrant extract has also been shown for 2 × 4 km cycling time trials with 10 min active recovery between trials in male cyclists (*n* = 10, age: 30 ± 12 years, $\dot{V}O_{\text{2max}}$: 55 ± 7 mL·kg^−1^·min^−1^) (105 mg anthocyanin·day^−1^ for 7 days) with a 0.82% faster overall time (19). In Murphy et al. (19), participants had only one full familiarization, but similar to Cook et al. (15), testing was in morning sessions after a light breakfast and without dietary restrictions during the 7-days dosing period. As far as we know, no studies with dark-colored berries other than blackcurrant have shown significant effects to enhance cycling performance. However, Nieman et al. (20) reported on 75-km cycling time trial performance with 2 weeks intake of powder made from low-bush wild blueberries (*Vaccinium angustifolium*) (26 g·day^−1^, TAC was 345 mg cyanidin-3-*O*-glucoside equivalent) (water: 195 ± 8.5 min vs. blueberry: 178 ± 6.6 min) in a study with polyphenol restricted intake 3 days before testing after an overnight fast. The study by Nieman et al. (20) had a parallel mixed-group design with four conditions (potentially reducing statistical power to show a significant 8.7% change). Blueberry contains ~24.4% delphinidins of TAC [http://phenol-explorer.eu/: (7)].

For running performance, Perkins et al. (21) used a randomized, placebo-controlled, double-blind, cross-over design and reported for the first time a berry-induced performance-enhancing effect for high intensity intermittent treadmill running of 10.6% for total distance (placebo: 3,871 ± 622 m; New Zealand blackcurrant extract: 4,282 ± 833 m). Similar to Cook et al. (15), participants were tested in a morning session, were allowed a small breakfast 3 h before testing and did not need to adhere to dietary restrictions during the 7 days of dosing. In addition, the dosing strategy was similar to Cook et al. (15) with 7-days intake of capsulated extract made from New Zealand blackcurrants (105 mg anthocyanins·day^−1^) with the last dose 2 h before testing. Participants performed one full familiarization (210. For running performance in field testing, New Zealand blackcurrant extract (105 mg anthocyanins·day^−1^ for 7 days), reduced slowing of the fastest sprint between block 1 and 5 (PL: 0.12 ± 0.07 s; New Zealand blackcurrant extract: 0.06 ± 0.12 s; *p* < 0.05) in the Loughborough Intermittent Shuttle Test (LIST) in recreationally active males (*n* = 13, age: 22 ± 1 years, $\dot{V}O_{\text{2max}}$: 50 ± 5 mL·kg^−1^·min^−1^) (22). In this study, the familiarization consisted only of 2 × 15 min blocks of the LIST. The effectiveness of New Zealand blackcurrant extract was more apparent in the later stages of the LIST (i.e., stage 4 and 5), suggesting an ergogenic effect while being in a fatigued state (22) and experiencing oxidative stress-induced fatigue (23). In addition, New Zealand blackcurrant extract (210 mg anthocyanins·day^−1^ for 7 days) had the ergogenic effect of reduced slowing of sprint time in the running-based anaerobic sprint test (6 × 35-m maximal sprints interspersed by 10-s passive recovery periods) later in the protocol (i.e., sprint 5) (24). The observations by Willems et al. (22) and Godwin et al. (24) rationalizes future work with experimental designs that incorporate standardized exercise-induced fatiguing protocols to examine the effectiveness of berry supplements on subsequent exercise performance and physiological responses. A recent study by Pastellidou et al. (25) reported for recreationally active males (*n* = 15, age: 24.4 ± 3.6 years) a trend for a performance-enhancing effect for running to exhaustion at a critical speed (*p* = 0.08) and at 110% of $\dot{V}O_{\text{2max}}$-speed (*p* = 0.09) test with intake of New Zealand blackcurrant extract (105 mg anthocyanins·day^−1^). Participants in Pastellidou et al. (25) did not seem to have been familiarized for the performance tests, had breakfast (no details on intake time and content) and there were no dietary restrictions. In addition, there was daily dosing over a period of weeks and it is unclear what the history of dosing was with observation of a trend for running performance (25). A recent study with the cyanidin-3-*O*-glucoside-rich Haskap berry [79–92% of TAC (26)] in male recreational runners provided faster 5 km time trial performance by 1.6% (27). This study had a double-blind, placebo-controlled, independent group design with restricted polyphenol intake during the 6-day dosing (~150 mg anthocyanins·dose^−1^) and final doses ~3 and 1 h before the 5 km time trial (27). Performance-enhancing effects by intake of New Zealand blackcurrant extract were not always observed. No effect was observed for a 16.1 km cycling time trial during normobaric hypoxia in male cyclists (morning session, 210 mg anthocyanins·day^−1^) (28), and in male cyclists in a fed state (afternoon session, 105 and 210 mg anthocyanins·day^−1^ (29), both studies with 7-day intake. These observations may suggest that the effectiveness of New Zealand blackcurrant extract with either 105 or 210 mg of anthocyanins are time-of-day and environment dependent. The inconsistency of observations will inform the future directions that are suggested for research on the effects of anthocyanin-rich supplementation.

## Future Directions For Sport And Exercise Nutrition Research With Anthocyanin-Rich Supplementation

Since the early studies on now established sport supplements [e.g., creatine: (30)], the discovery of the ergogenic potential and factors that influence effectiveness are still ongoing [e.g., timing of creatine supplementation on hypertrophy during resistance training: (31)]. Decades of discovery for anthocyanin-rich supplementation in the field of sport and exercise nutrition have just started. Most sport and exercise performance supplementation studies employ the *gold standard* randomized, double-blind, placebo-controlled cross-over design, with a few exceptions of studies with testing more than once in the placebo and supplementation condition [e.g., caffeine: (32), sodium bicarbonate: (33, 34)]. Individual responses for physiological and performance responses are commonly ignored [e.g., beetroot: (35), caffeine: (36)]. With respect to anthocyanin intake, the landmark study by Czank et al. (37) on the absorption, distribution, metabolism and elimination of isotopically labeled cyanidin-3-*O*-glucoside (6,8,10,3′,5′-^13^C_5_-C3G) noted the interindividual variability in total ^13^C recovery from urine, fecal, breath and blood samples with a range from 15.1 to 99.3% over a 2-day period. The interindividual variability was present after an overnight fast and avoidance of anthocyanin-rich foods and food with a higher natural abundance of ^13^C 7-days before and during the 48-hr study period (37). Some anthocyanin-induced metabolites (e.g., hippuric acid) are still present more than 24 h after intake of cyanidin-3-glucoside (37). Costello et al. (38) showed that acute intake of New Zealand blackcurrant extract (anthocyanin content 105 mg) following an overnight fast in 29 participants (age: 28 ± 7 years, nine females) increased the plasma uptake of phenolic acids gallic acid and protocatechuic acid but not vanillic acid. This effect was apparent even when a habitual diet was maintained in the days preceding the experimental trail (i.e., polyphenols were not retracted) and similar to Czank et al. (37), inter-individual variability between participants was apparent. The reproducibility of the interindividual bioavailability for consumed anthocyanins and the numerous anthocyanin-induced metabolites is not known. In addition, a loading phase (i.e., daily anthocyanin intake) is required to change some physiological responses [e.g., walking-induced fat oxidation: (39)], thus daily bioavailability and the reproducibility needs to be known to assess potential accumulation of anthocyanin-induced metabolites. Accumulation of anthocyanin-induced metabolites in blood over a long period may be possible [e.g., protocatechuic acid-3-O-sulfate by 16 weeks daily intake of 320 mg blackcurrant and bilberry anthocyanins: (40)]. Important is to establish a potential causal link between the bioavailability of anthocyanins and anthocyanin-induced metabolites and exercise performance, the reproducibility of the bioavailability and the impact on exercise performance, and identifying responders and non-responders. Our understanding on the effectiveness and the mechanisms of anthocyanin supplementation would benefit from studies with block randomization design with replication (41), a design in which participants are tested twice for the placebo and the nutritional condition. Such studies would allow identification of repeated responders using proposed statistical frameworks (42). In addition, studies should always have full familiarizations to establish a stable baseline (43).

Studies with anthocyanin intake with implications for athletes have not had the intake duration as is common for clinical studies [e.g., 3 months tart cherry: (44); 6 months blueberry: (45)]. For normobaric hypoxia and fed state conditions (28, 29), future studies are needed to examine the effects of intake duration and higher doses of anthocyanins on exercise performance. Higher doses of anthocyanins are not uncommon in clinical studies, e.g., 724 mg blueberry anthocyanins on vascular function and neutrophil NADPH oxidase activity in healthy 18–40 year old men (*n* = 21) (46). For the studies with short intake duration of anthocyanins [e.g., (15, 47)], it is still unknown how the bioavailability of anthocyanin-induced metabolites is associated with the observed physiological, metabolic and cardiovascular responses. For an athlete training over periods of months for performance enhancement, future studies with consistent longer intake of anthocyanins during a physical training program should examine whether there is an enhanced training effect. Animal studies have shown, for example, that mouse fed with bilberry and blackcurrant anthocyanins for 4 weeks (48) and with the blackcurrant anthocyanin cyanidin-3-glucoside for 14 days (49), promoted expression of expression of peroxisome proliferator-activated receptor-gamma coactivator-1α (PGC1-α). PGC1-α is a positive regulator of the process to increase the number of mitochondria, a key objective adaptation of moderate-intensity continuous training. Future work should examine the additive effects of anthocyanin-rich supplementation on the adaptations by physical training modalities and address concerns that the antioxidant effects may blunt training adaptations. Adaptations for PGC1-α and the mitochondrial marker cytochrome c oxidase subunit IV, for example, were blunted in recreationally endurance trained individuals (*n* = 27, 14 women) following daily high intake of capsulated vitamins C and E (500 and 117 mg·day^−1^) during a 11-week endurance training program (50). PGC1-α was also blunted by high intake of vitamins C and E (1,000 mg·day^−1^ and 400 IU·day^−1^) during a 4-week physical intervention in males (51). In addition, confirmation of the need of oxidative stress for endurance training adaptations was provided by Margaritelis et al. (52) with participants experiencing low exercise-induced oxidative stress (cycling at 70% of maximum power) showing lowest improvements. Training studies with daily blackcurrant intake would have high ecological validity and provide potentially observations on at least non-blunting training adaptations that would inform dosing strategies for athletes in preparation of competition. Such studies can also address the effect of months of intake of anthocyanins on the diversity of the gut microbiota, as just anthocyanin intake can alter the gut microbe diversity [e.g., black raspberry in a human colonic model: (53), blueberry for 6 weeks in fecal microbiota in healthy older but not younger women: (54)]. When months of intake of anthocyanins change the gut microbe diversity in athletes, this may affect also the bioavailability of anthocyanins and anthocyanin-induced metabolites due to the conversion role of the gut microbes and contributing to the bioavailability.

## Concluding Remarks

In the field of sport and exercise nutrition, most studies that have used anthocyanin-rich New Zealand blackcurrant extract have shown meaningful effects on exercise performance. However, the anthocyanin profile and the dosing strategy (i.e., intake duration, timing, and dose) that provides optimum benefits in preparation for and competing in athletic events for athletes with different training status and habitual dietary intake is not known. The diversity of the anthocyanin profile of berries and the physical (and cognitive) demands of sports will justify the continuation of a broad research agenda to examine the practical applications and mechanisms of effect of anthocyanin-rich supplementation in the field of sport and exercise nutrition. Findings from studies with New Zealand blackcurrant extract promise strong potential for anthocyanin-rich supplementation to become an established ergogenic nutritional aid for a wide range of athletic activities, athletes with different training status and physically active individuals.

## Data Availability Statement

The original contributions presented in the study are included in the article, further inquiries can be directed to the corresponding author.

## Author Contributions

MW prepared the initial draft. SB edited and revised the article. Both authors approved the manuscript.

## Funding

This work was funded by Blackcurrants New Zealand Inc. (https://www.blackcurrant.co.nz/). The funder was not involved in the content of the perspective, the interpretation of the data, the writing of this article or the decision to submit it for publication.

## Conflict of Interest

The authors declare that the research was conducted in the absence of any commercial or financial relationships that could be construed as a potential conflict of interest.

## Publisher's Note

All claims expressed in this article are solely those of the authors and do not necessarily represent those of their affiliated organizations, or those of the publisher, the editors and the reviewers. Any product that may be evaluated in this article, or claim that may be made by its manufacturer, is not guaranteed or endorsed by the publisher.
